# The basal forebrain modulates spontaneous activity of principal cells in the main olfactory bulb of anesthetized mice

**DOI:** 10.3389/fncir.2013.00148

**Published:** 2013-09-20

**Authors:** Xiping Zhan, Pingbo Yin, Thomas Heinbockel

**Affiliations:** ^1^Department of Anatomy and Neurobiology, University of Maryland School of MedicineBaltimore, MD, USA; ^2^Department of Physiology and Biophysics, Howard University College of MedicineWashington, DC, USA; ^3^Department of Electrical and Computer Engineering, Institute for Systems Research, University of MarylandCollege Park, MD, USA; ^4^Department of Anatomy, Howard University College of MedicineWashington, DC, USA

**Keywords:** mitral cell, tufted cell, diagonal band, synchronization, scopolamine, olfactory bulb

## Abstract

Spontaneous activity is an important characteristic of the principal cells in the main olfactory bulb (MOB) for encoding odor information, which is modulated by the basal forebrain. Cholinergic activation has been reported to inhibit all major neuron types in the MOB. In this study, the effect of diagonal band (NDB) stimulation on mitral/tufted (M/T) cell spontaneous activity was examined in anesthetized mice. NDB stimulation increased spontaneous activity in 66 MOB neurons which lasted for 2–35 s before returning to the baseline level. The majority of the effected units showed a decrease of interspike intervals (ISI) at a range of 8–25 ms. Fifty-two percent of NDB stimulation responsive units showed intrinsic rhythmical bursting, which was enhanced temporarily by NDB stimulation, whereas the remaining non-rhythmic units were capable of synchronized bursting. The effect was attenuated by scopolamine in 21 of 27 units tested. Only four NDB units were inhibited by NDB stimulation, an inhibition that lasted less than 10 s. The NDB stimulation responsive neurons appeared to be M/T cells. Our findings demonstrate an NDB excitation effect on M/T neurons that mostly requires muscarinic receptor activation, and is likely due to non-selectivity of electrical stimulation. This suggests that cholinergic and a diverse group of non-cholinergic neurons in the basal forebrain co-ordinately modulate the dynamics of M/T cell spontaneous activity, which is fundamental for odor representation and attentional perception.

## Introduction

The main olfactory bulb (MOB) is a unique relay station that gates transmission of olfactory receptor signals to higher brain areas (Kay and Sherman, 2007), where these signals are integrated and modulated (Mandairon and Linster, 2009). Mitral/tufted cells (M/T) in the MOB receive inputs to their dendrites in the glomeruli from olfactory receptor neurons (ORN), and in turn M/T cells project their axons to olfactory cortical and limbic structures. A majority of these cells discharges spontaneously even when olfactory nerve transmission is blocked, which is fundamental for odor representation (Stakic et al., 2011). As ORNs are receiving a multitude of stimuli, input signals from ORNs to M/T cells add to noise activity of M/T cell. Hence, the signal-to-noise ratio is critical for M/T cell representation of odor signals, notwithstanding that some neurons are rate-invariant that may use another mechanism (Gschwend et al., 2012). M/T cells not only receive excitatory input in one glomerulus, they also receive inhibitory input from neighboring glomeruli through inhibitory interneurons located in the glomerular layer or external plexiform layer (Wachowiak and Shipley, 2006; Wilson and Mainen, 2006). Thus, the signal-to-noise ratio of M/T cells can be modulated by the intrinsic circuits within the bulb. Furthermore, these intrinsic circuits are also modulated by the higher brain structures, including the basal forebrain (Inokuchi et al., 1987; Nickell and Shipley, 1988; Kunze et al., 1992; Ma and Luo, 2012).

The basal forebrain has been suggested a major centrifugal modulation center, which has diverse projections to the neocortex and plays an essential role in attention and cognitive functions (Sarter et al., 1999; Conner et al., 2003). This structure contains the Nucleus of Diagonal Band of Broca (NDB), which is composed of a Vertical Diagonal Band (VDB), and a Horizontal Diagonal Band (HDB). Neurons in the HDB have considerable GABAergic and cholinergic projections to the MOB (Kasa et al., 1995, 1996; Gracia-Llanes et al., 2010). Periglomerular cells (PG) and granule cells, respectively, are two major types of cholinoreceptive cells located in the glomerular and inframitral layers (Kasa et al., 1995, 1996; Castillo et al., 1999). Muscarinic acetylcholine receptors (mAChRs) and nicotinic acetylcholine receptors (nAChRs) are distributed in the inframitral layer and glomeruli with little overlap (Le Jeune et al., 1995). Previous *in vitro* work suggests exclusive extrinsic cholinergic modulation of M/T cell function, given that HDB cells are the only source of cholinergic input to the MOB (Mesulam et al., 1983; Fletcher and Chen, 2010). However, local intrinsic cholinergic activity has also been described (Phelps et al., 1992; Krosnowski et al., 2012). NDB modulation of M/T unit activities has been examined in a few studies (Inokuchi et al., 1987; Nickell and Shipley, 1988; Kunze et al., 1991), in which the effect on spontaneous activity was contradictory. More recently, Ma and Luo (2012) revealed that activation of cholinergic neurons from HDB inhibits exclusively the major types of MOB neurons. However, cholinergic neurons only constitute about 20% of HDB (Shipley et al., 2003), and about half of the basal forebrain neurons which project to the cortex are GABAergic neurons (Sarter and Bruno, 2002). Here, we have investigated how NDB modulates spontaneous activity of MOB principal neurons with electrical stimulation, and found that most of the presumable M/T cells are excited by NDB stimulation and that muscarinic receptors are required. The effect suggests a diversity of modulation on M/T neuron spontaneous activity.

## Materials and methods

### Surgical preparations

The experiments were performed in adult (3- to 4-month old), male C57BL/6J mice (*n* = 137), 25–32 g, anesthetized with urethane (IP, 1.5 mg/kg). Levels of anesthetics were adjusted to maintain surgical anesthesia as judged by depth and rate of respiration (1–4 Hz), as well as presence of a moderate foot withdrawal reflex. The animals were then placed in a stereotaxic device for surgery. An insulated peltier warming plateform was used below the animal to monitor thermoregulation. Body temperature was maintained at 35–37°C. The respiration was monitored and recorded with a Plexon System (Dallas, TX). The skin was cut and spread to expose the skull above the MOB and the cortex overlying a diagonal band.

### Extracellular single unit recording

**Figure 1:**
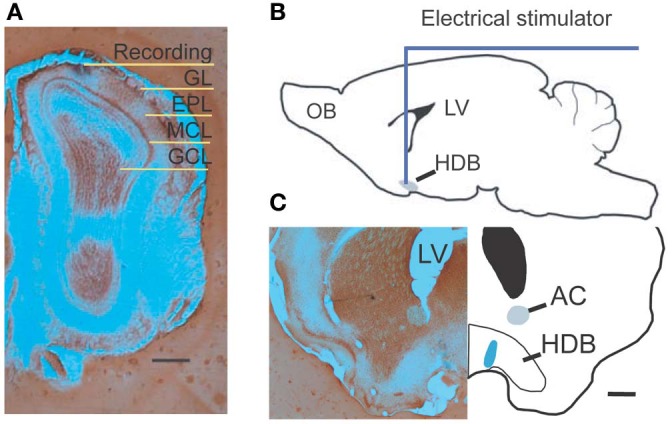
**Experimental diagram. (A)** Photograph shows a recording location with pontamine sky blue located in the superficial external plexiform layer. **(B)** A drawing shows the location of NDB stimulation; the HDB is highlighted. **(C)** An examplar stimulating location shows blue ion deposits from stimulating electrode visualized by potassium ferrocyanide. Scale bar = 250 μm.

Extracellular recordings were performed with glass capillary pipettes filled with 1 M NaCl, or 0.5 M NaAc with 2% pontamine sky blue (Sigma, MO), or FHC tungsten microelectrodes. Electrode impedances were in the range of 1–4 MΩ. The electrode was approaching the dorsal medial olfactory bulb with an angle of 45° in mid-sagittal plane, and ranged from 20 to 350 μm below the surface of the olfactory bulb (Figure 1A). The real-time spike activity and local field potentials (LFPs) were recorded by Plexon System (Dallas, TX). Signals from the recording electrode were amplified 20× by an op-amp headstage (Plexon, HST/8o50-G20-GR), and were further passed through a differential preamplifier (Plexon, PBX2/16SP-R/16FP-G50), where they were amplified 50×. The spike unit signals were extracted by being filtered online between 150 and 10 kHz. The single unit signals were then sent to the Multichannel Acquisition Processor box, where they were digitized at 20 kHz and amplified at 1–20×.

### Electrical stimulation and drug delivery

A bipolar concentric stimulating electrode was lowered into the NDB based on the coordinates determined from the stereotaxic atlas (Figure 1B) (Paxinos and Franklin, 1997). The tip diameter of the microelectrode was 125 μm, and it had an impedance of 200–500 kΩ. Optimal placement was determined if there was a stimulation response when recording was ongoing in the MOB. If a unit had casual or no spontaneous activity, the recording was discontinued. All stimulating currents were delivered by a constant current stimulus isolation unit. A train of 25 electric pulses was applied with a few exceptions, which was delivered at 100 Hz (Cygnus, PG4000A). Each stimulation had a duration of 100 μ s, and a magnitude of 300–500 μ A. NDB stimulation-induced increases or depressions of spiking were determined by comparing maximal post-stimulus spike firing rate to the pre-stimulus spontaneous spike rate, and by verifying that the relevant interspike interval (ISI) was altered significantly. The test time window was set to 5 s before or after electrical stimulation. For each trial, the neuronal spiking activity was recorded for 60 s unless otherwise stated. When trials were repeated for more than 10 min with stable spontaneous activity and an NDB stimulation-induced effect, delivery of scopolamine (Sigma, St Louis, MO), a non-selective mAChR antagonist was started. After drug delivery, the trials were repeated for 30 min to 2 h. An IP injection of 5–7.5 mg/kg scopolamine was dissolved in 0.9% NaCl. Alternatively, in order to apply scopolamine by dripping it on the surface of the olfactory bulb, scopolamine was dissolved in artificial cerebrospinal fluid (300–600 μ M), and delivered by a disposable pipette in a total volume of 20 μ l. After recording, a continuous current of 20 s at a strength of 50 μ A was applied for deposition of iron; alternatively, a higher current of 1 mA was used to induce a lesion in the stimulating location (Figure 1C). Animals were perfused immediately afterwards, with 0.9% saline followed by 1% potassium ferrocyanide, and routine histological processing thereafter (Figure 1C).

### Data analysis

The individual units were identified and isolated by a clustering method with Offline Sorter 2.8.8 software (Dallas, TX) using a template-matching algorithm, and a tolerance was set at 90%. Waveform clusters were considered from the same neuron, and 1% of the neurons have an ISI <2 ms. Following sorting, unit waveforms were manually verified in terms of their amplitude consistency across trials. Sorted files were then processed in NeuroExplorer (Littleton, MA) to extract unit timestamps and relevant event markers. Further analysis was performed with Matlab (The Mathworks, Natick, MA). Peri-stimulus time histograms were made from 10 to 12 trials and binned of 0.5 s. For respiration triggered histogram, the time component of respiration in each trial was extracted and converted into [−π 0] and [0 π]. The recording spikes were aligned to the transition point (0) between inhalation and exhalation. The respiration traces were normalized from relevant trials (Figures 3A, 4A, lower panels). The significance of changes of spiking rates was evaluated with Student's *t*-test, whereas Kolmogorov–Smirnov test were performed for all ISI measurements.

## Results

**Figure 2:**
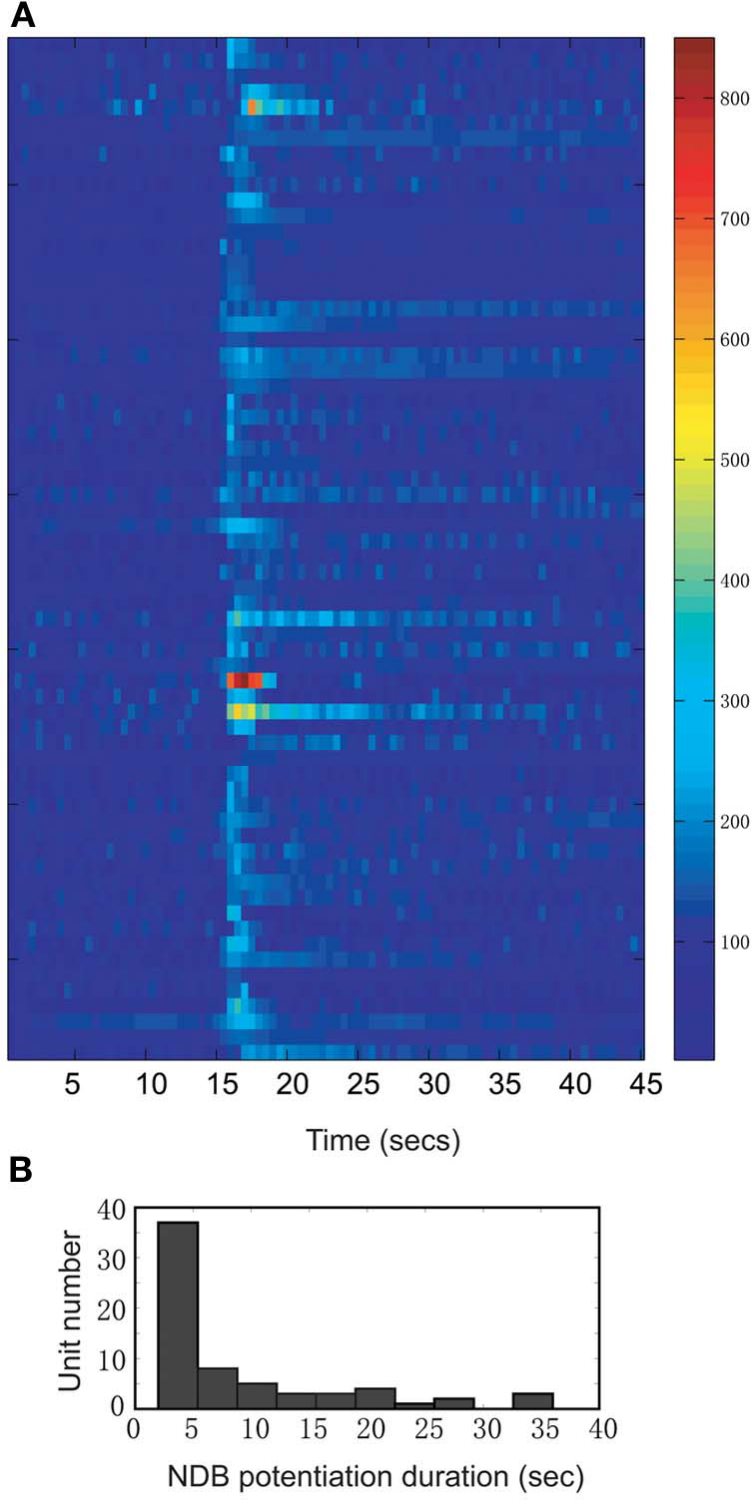
**A summary of all units showing increased firing rate following electrical stimulation (*n* = 66; Bin = 0.5 s). (A)** The time course records are normalized to the baseline period 12–15 s. The color bar on the right indicates the normalized spike rates (%). **(B)** Distribution of NDB potentiation shows the range of duration from 2 to 35 s.

We investigated the neuronal response to NDB stimulation in the MOB of 41 anesthetized mice. Histological work afterwards confirmed the stimulations were located in the NDB (Figure 1C). Sixty-six units showed increased spontaneous activity (hereafter referred to as NDB potentiation) to NDB electrical stimulation (Figure 2A), representing about 17% of the total MOB units identified. The NDB responsive units had a baseline firing rate ranging from 2 to 31 Hz. For 57 of the 66 units, 25 stimulus trains were used to induce increased response, whereas for the other 9 units, more stimulus trains were used (from 30 to 150 pulses). Out of the total 70 units responsible to NDB stimulation, four units (6%) showed decreased spike rate following NDB stimulation (referred to as NDB suppression, Figure 6). Based on the pontamine sky blue labeling, the recording sites were in a range equivalent to the mitral cell layer and the external plexiform layer (Figure 1A). In our preparation, granule cells discharged with much smaller amplitudes or did not appear to discharge spontaneously, therefore spontaneous activity combined with spike amplitude, firing pattern, as well as electrical stimulation-induced LFPs were used as physiological criteria to localize recording electrodes (Kay and Laurent, 1999; Davison and Katz, 2007; Bathellier et al., 2008). In our recordings, NDB responsive neurons were identified as M/T cells. All electrically induced LFPs appeared as a positive wave peak followed with a negative trough (data not shown), which is typical in the mitral cell layer with NDB electrical stimulation (Nickell and Shipley, 1988).

### Characteristics of the NDB stimulation effects in the MOB

Following electrical stimulation, the NDB spiking potentiation generally persisted for 2–5 s, but in some cases lasted as long as 35 s before returning to the level of baseline spontaneous activity (Figure 2). In 56% of the units the firing rate returned to the control within 5 s, whereas 28% of them recovered in 5 to 20 s, and the other 16% of the units recovered after more than 20 s (Figure 2B; *n* = 57: stimulation, 100 Hz, 25 pulses). The long-lasting NDB potentiation effect observed here is an indication that the NDB can potentially affect M/T cell spontaneous activity in a much wider temporal window.

**Figure 3:**
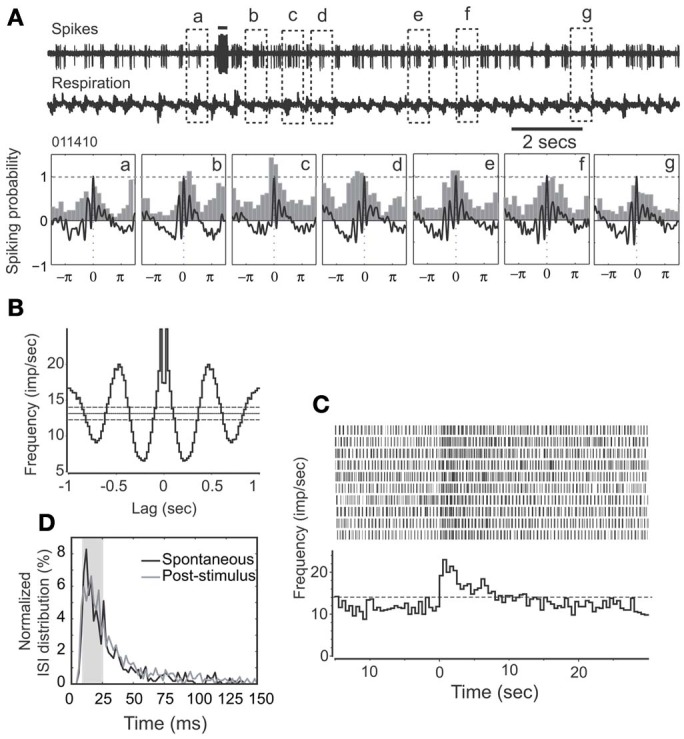
**HDB stimulation does not affect synchronization pattern of a rhythmically spiking unit. (A)** Raw traces show spikes and respiration recorded simultaneously. Note that the unit remains synchronized with respiration following HDB stimulation. This unit has a long-lasting discharge activity. Lower panel: The respiration triggered spike histograms are shown before (a) or after stimulation (b–g). Respiration traces are averaged and 0 stands for the transition point between inhalation and exhalation. The top bar: electrical stimulation (100 μs, 400 μA, 100 Hz, 25 pulses). **(B)** An autocorrelation analysis indicates the unit has a rhythmicity of 3 Hz at a confidence limit of 99% (bin = 20 ms). **(C)** Histogram and raster plot showing stimulation effects on mean spike rates. Note *t* = 0 indicates the beginning of NDB stimulation, and this applies to the rest of Figures. **(D)** Plot showing ISI distributions before or after stimulation, respectively. Note that ISIs following NDB stimulation are decreased (*p* < 0.001).

**Figure 4:**
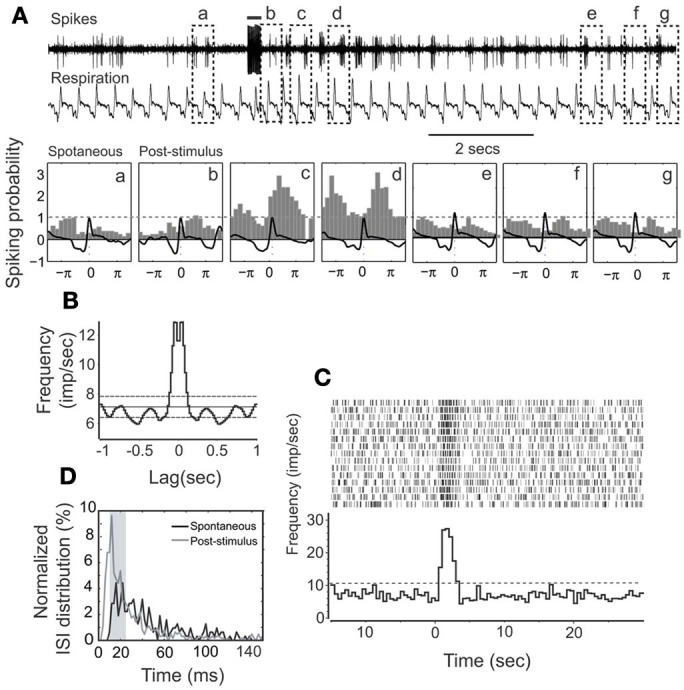
**HDB stimulation on a non-synchronized, rhythmically spiking unit. (A)** Raw traces show the simultaneous recording of spikes and respiration. This unit has a short-lasting discharging activity. The bar on the top shows the electrical stimulation (100 μ s, 500 μ A, 100 Hz, 25 pulses). The respiration triggered spike histograms are shown before (a) or after stimulation (b–g) as described in Figure 3. **(B)** An autocorrelation analysis indicates the unit rhythmicity (Bin = 20 ms). **(C)** Histogram and raster plot showing potentiation of spike rates. **(D)** Plot showing ISI distributions before or after stimulation, respectively. Note that ISIs following NDB stimulation are decreased (*p* < 0.001).

Cellular activity that is synchronized with respiration is a typical property of M/T cells and critically involves intrinsic MOB circuits (Buonviso et al., 2006). Among NDB stimulation responsive neurons, about 52% (34/66) showed rhythmic bursting synchronized with respiration at frequencies of 1–4 Hz (Figure 3B); the remaining 48% (32/66) exhibited non-synchronized firing (Figure 4B). For all the units with increased spiking activities (Figures 3C, 4C) the change in mean spike rate was confirmed by a reduction of ISIs to a range of 8–25 ms (Figures 3D, 4D). For the non-synchronized or less salient neurons, spiking activity can be induced to be time locked with respiration (Figure 4A). For those synchronized bursting units, even though the mean spike rates were elevated, the bursting patterns were not altered (Figure 3A). This applies to the decay phase of the long-lasting NDB potentiation, suggesting a physiological property shared by long-lasting and short NDB responses. The NDB potentiation achieved its maximal effect in the third or fourth rhythmic circle (Figures 3A, 4A). For some long-lasting units, there was an extra band, ranging from 2 to 10 ms, an indication of unknown faster events (Figure 8C; *n* = 3).

Neuronal spontaneous activity is dynamic in different functional or behaving states. We used it to plot NDB stimulation-induced response strength (*Rs*) to determine the relationship between the pre-stimulus spontaneous spike rate and NDB stimulation responsiveness. The NDB stimulation-induced response strength is given below:

$$Rs=\frac{j}{i}\frac{\sum Ni}{\sum Nj}$$

**Figure 5:**
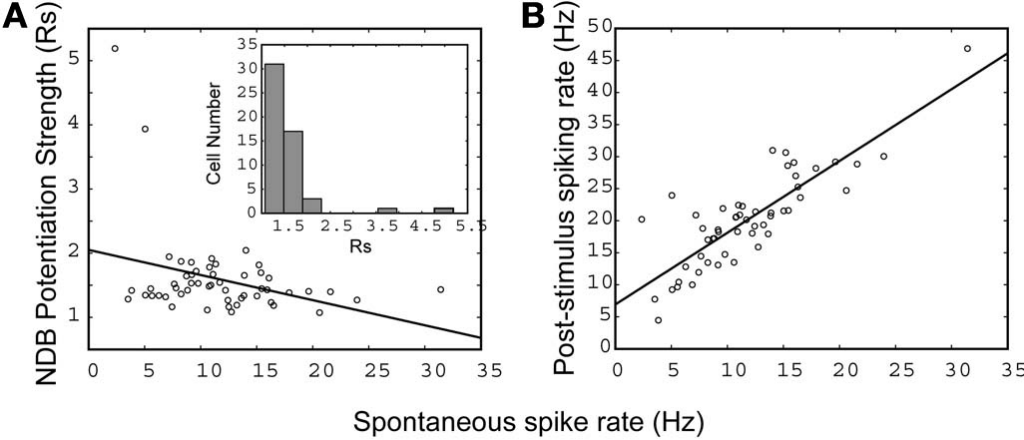
**The relationship between spontaneous activity and NDB potentiation strength (A), and post-stimulus activity (B).** A linear fit is applied and the correlation coefficient is indicated separately: **(A)** *r* = −0.38, **(B)** *r* = 0.89 (*n* = 57). (**A**, inset), a histogram shows the distribution of cells with NDB stimulation-induced response strength (Rs).

*Ni* and *Nj* are average post-stimulus spike rates at bin *j* and average pre-stimulus spike rates at bin *i*, respectively (bin = 0.5 s). Accordingly, *Nj* and *Ni* are estimated 5 s prior to stimulation onset and 5 s after. Based on a linear fitting, there was a negative correlation between the pre-stimulus spontaneous activity and the NDB stimulation-induced response strength (*r* = −0.38; Figure 5). This suggested that the NDB potentiation was inversely correlated to M/T cell basal spike rate, given that neurons had a saturable upper limit. Combined with the characteristics of diverse oscillation described above, M/T neurons have heterogeneous biophysical properties in terms of their responsiveness to NDB electrical stimulation, which is consistent with previous observations (Padmanabhan and Urban, 2010; Angelo and Margrie, 2011). As M/T cells in wake, behaving animals have lower spontaneous activity that may be changed in different functional or behavioral states we postulate that NDB may modulate M/T cell activity accordingly. We further plotted the average pre-stimulus spike rate as a function of the average response, and found a simple linear correlation (*r* = 0.89, Figure 5B). The majority of cells had an *Rs* range of 1.3–2 with few exceptions (Figure 5A, inset). This implies that more weakly firing principal cells have a more dynamic range to be potentiated by NDB activity.

**Figure 6:**
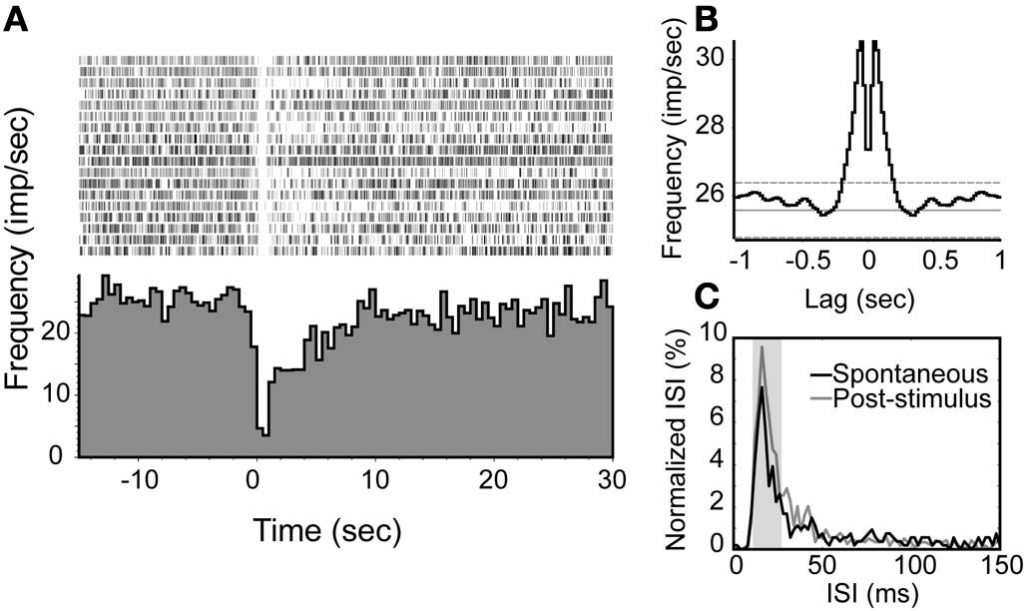
**An examplar unit with an inhibition response in which the spiking activity is decreased transiently following NDB stimulation. (A)** Peri-stimulus histogram and raster plot show the decreased spiking with an average of spontaneous spike rate at 26 spikes/s. **(B)** Autocorrelation of spontaneous spikes. Bin = 20 ms. **(C)** The ISIs of this unit are elevated at 10–25 ms.

For depressed NDB units, the neurons had spontaneous activity rates ranging from 10 to 26 spikes/s and they were non-rhythmic (Figure 6B). The suppression was shorter, and persisted for about 2–10 s (Figure 6A). This was confirmed by a marked shift of ISIs in the range of 10–25 ms (*p* < 0.001, Figure 6C).

### Muscarinic acetylcholine receptors contribute to NDB potentiation

Several studies have revealed that muscarinic receptors exert multiple and opposing functional effects in the MOB (Castillo et al., 1999; Ghatpande et al., 2006; Mandairon et al., 2006; Pressler et al., 2007; Chaudhury et al., 2009; Ghatpande and Gelperin, 2009). Earlier work using atropine indicated that it effectively blocked NDB-stimulation induced activity which is in conflict with findings obtained with optogenetic methods (Inokuchi et al., 1987; Ma and Luo, 2012). We re-examined this issue with scopolamine, a putative competitive mAChR antagonist.

**Figure 7:**
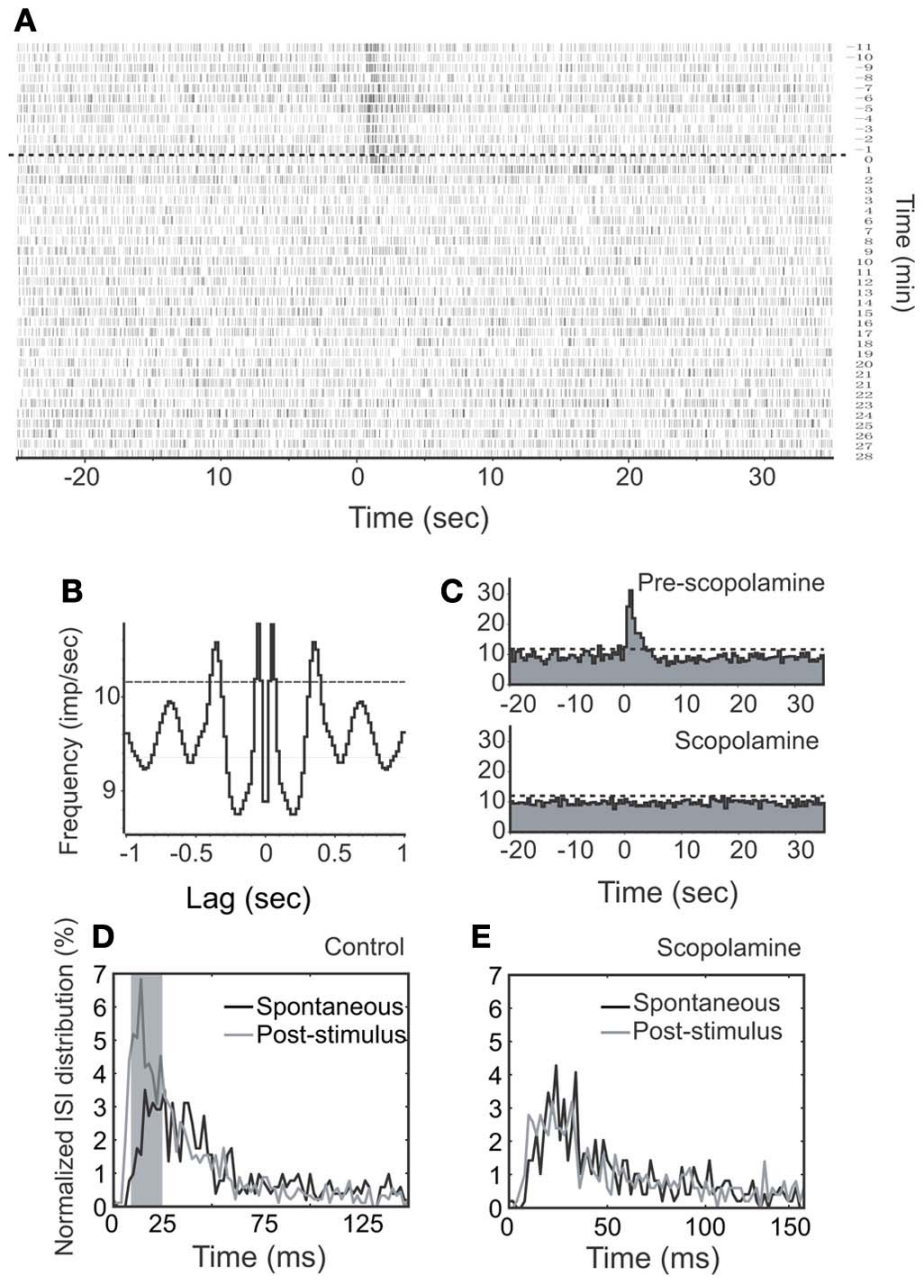
**Effects of stimulation and scopolamine on a respiration-synchronized rhythmic unit. (A)** A time course spiking recording shows the effects of NDB stimulation (100 μ s, 400 μ A, 100 Hz, 25 pulses) and scopolamine (IP, 5 mg/kg). Note this unit does not show recovery during the course of recording. The dashed line indicates the start time of scopolamine. **(B)** An autocorrelation analysis reveals the unit rhythmicity at 3 Hz (Bin = 20 ms). **(C)** Histogram of NDB-induced spike rates before and after scopolamine. **(D,E)** ISI distributions show effects before or after scopolamine treatments, respectively.

**Figure 8:**
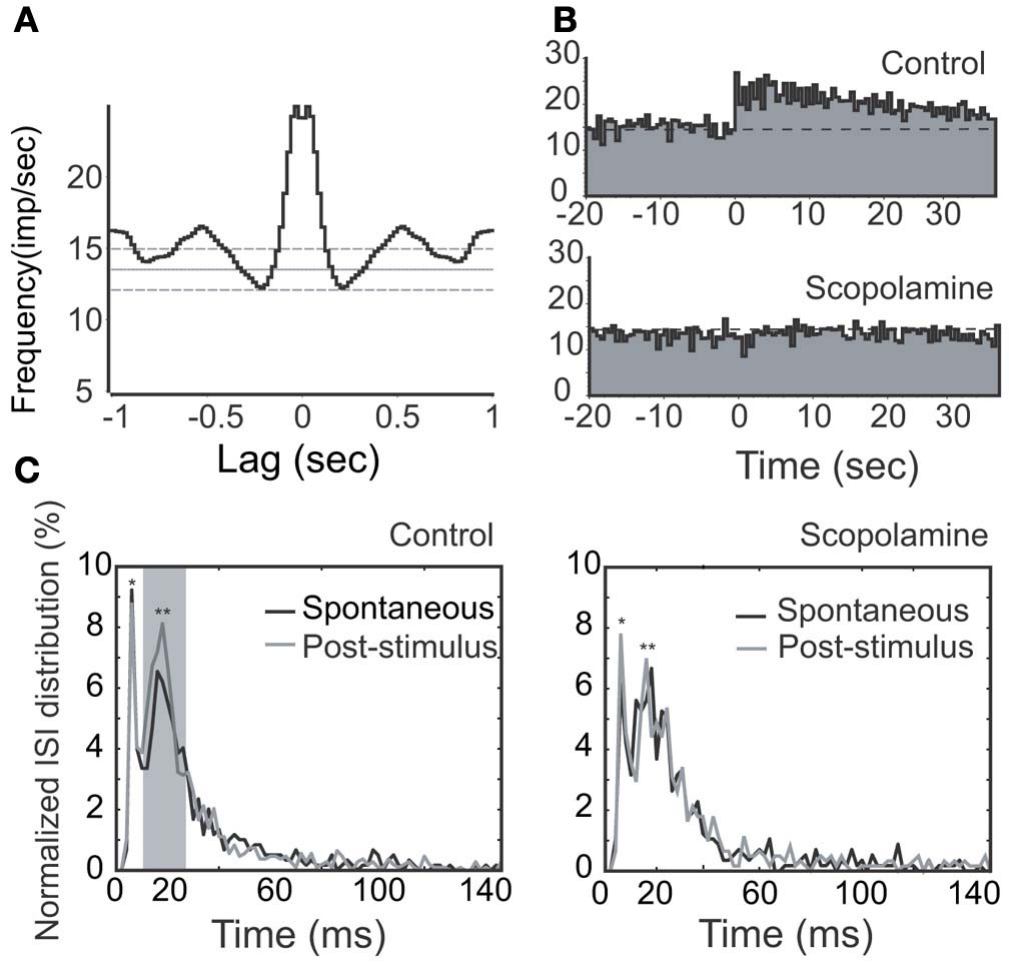
**Effects of stimulation (100 μs, 500 μA, 100 Hz, 25 pulses) and scopolamine on a rhythmic unit with long-lasting NDB potentiation. (A)** Autocorrelation of spontaneous spikes; Bin = 20 ms. **(B)** Histogram of NDB-induced spike rates before and after scopolamine administration (IP, 7.5 mg/kg). **(C)** ISI distributions are shown before (left) or after (right) scopolamine treatment, respectively. Note that this unit has a typical ISI range of 12–25 ms (^**^). It also has a faster characteristic spiking with an ISI range of 3–12 ms (^*^). Note that ISIs following NDB stimulation are decreased (*p* < 0.005), which is not changed significantly with scopolamine (*p* > 0.5).

We first injected scopolamine, a putative competitive mAChR antagonist into animals via IP (*n* = 7; Figures 7, 8), and found an attenuation of NDB stimulation-induced activity without a significant recovery (Figure 7A). The spontaneous discharge rates did not change significantly with a few exceptions (Figure 10A), while the NDB potentiation attenuated significantly. Scopolamine had pharmaceutical effects on diverse types of neurons when administered systemically, regardless of their characteristics of rhythmicity or NDB potentiation. Figures 7, 8 showed two synchronized rhythmic units, one with short- and one with long-lasting potentiation (Figures 7A,C, 8B). Both NDB stimulation-induced spike rates and ISIs were altered (Figures 7C,D, 8B,C). Scopolamine had an indistinguishable impact on the initial phase and decay phase for the long-lasting response (Figure 8B).

**Figure 9:**
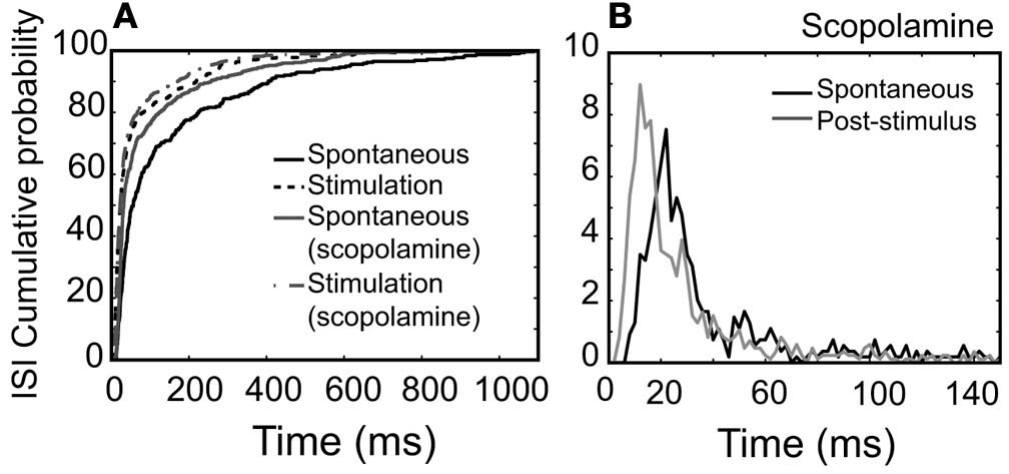
**NDB potentiation is not affected by scopolamine in a non-synchronized, rhythmically spiking unit (Figure 4).** For this unit, scopolamine was dripped at 600 μM. **(A)** Cumulative probability of ISI distributions in spontaneous or post-stimulus activity and the effect of scopolamine. **(B)** Plots showing ISI distributions after scopolamine treatments. Note that ISIs following NDB stimulation are decreased significantly (*p* < 0.001), which is not affected by scopolamine (*p* < 0.001).

**Figure 10:**
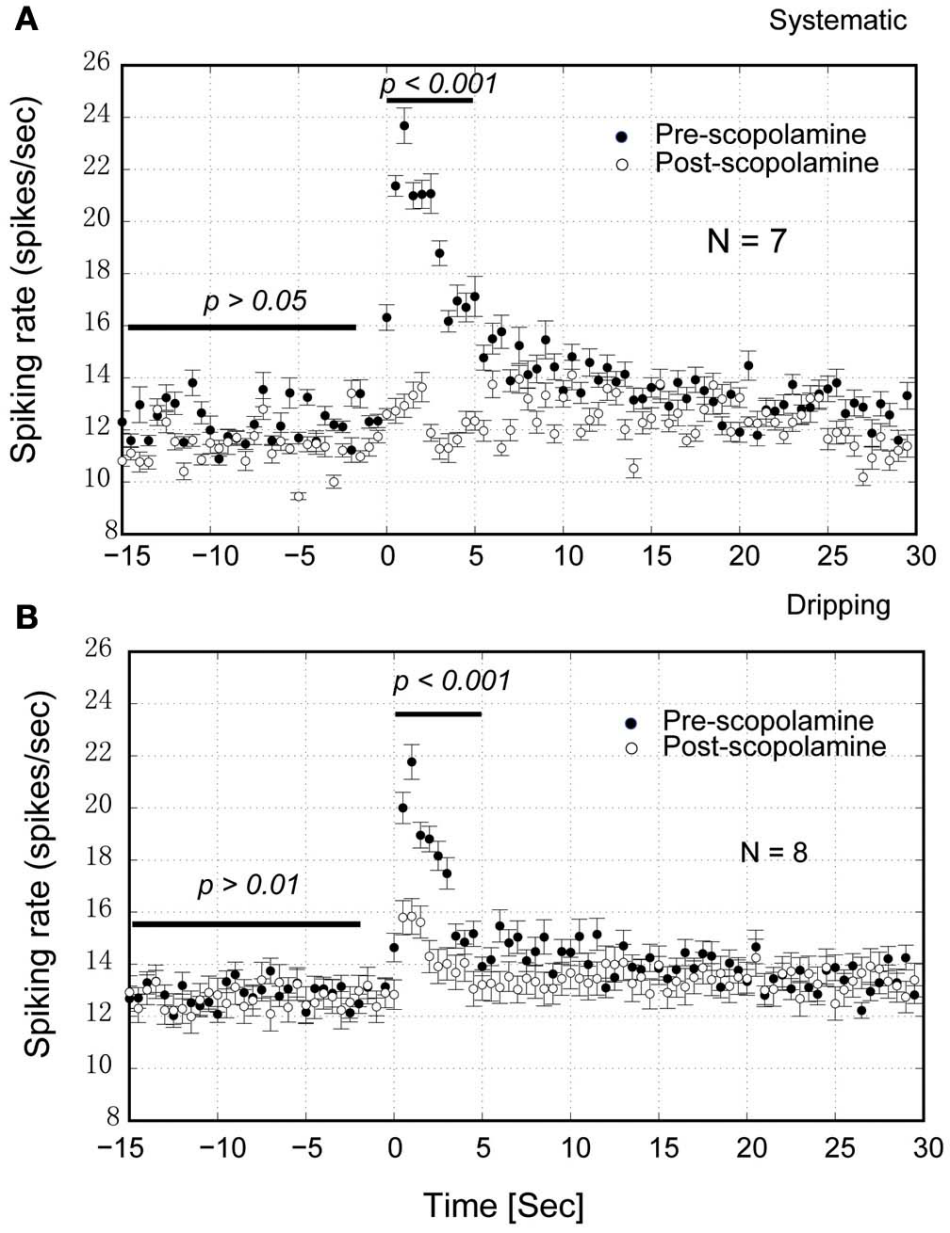
**Group statistics of the neurons show that scopolamine attenuates the NDB potentiation in the MOB. (A)** IP injection of scopolamine blocked the NDB potentiation completely (*p* < 0.001) but not the spontaneous activity (*p* > 0.05). **(B)** Dripping scopolamine on the top of MOB attenuated the NDB potentiation significantly (*p* < 0.001) without effect on the spontaneous activity (*p* > 0.01). Horizontal bar indicates the paired samples used for statistics.

To eliminate possible cholinergic involvement extrinsic to the MOB, we next dripped scopolamine directly onto the top of recording site (*n* = 14). In eight cases, NDB potentiation was attenuated significantly (*p* < 0.005, Figure 10B), but for six other cases, NDB potentiation was not significantly attenuated suggesting that the lack of an effect was not due to a lower concentration of scopolamine (*p* > 0.5; Figure 9). This implies that other non-muscarinic receptors may be involved; furthermore, it implies that the possible artifact caused by dripping or ACSF can be effectively excluded. Although different concentrations evoked varied effects, in all cases, the concentration of scopolamine was relatively high.

## Discussion

The HDB has been implicated in the modulation of MOB neuronal activity as revealed through single unit (Inokuchi et al., 1987; Kunze et al., 1991; Ma and Luo, 2012) and LFP recordings (Nickell and Shipley, 1988). We readdressed this issue with an electrical stimulation at higher frequencies, followed by examining effects on mean spike rates and patterns of spontaneous activity. We demonstrate that NDB electrical stimulation strongly boosted M/T cell spontaneous activity without affecting its rhythmic pattern, or even transformed non-synchronized bursting into synchronized oscillatory activity. Finally, for most of the NDB potentiation units, mAchRs were required.

### Consideration of electrical stimulation

Since the effective field of stimulation is determined by the excitability constant of stimulated neurons (Tehovnik, 1996), stronger currents might posit a spread of stimulation, causing artifacts which are not elicited from activation of NDB neurons alone. Of particular concern are the olfactory anterior commissural connections (Nickell and Shipley, 1993), fibers-of-passage from dorsal raphe nucleus (Petzold et al., 2009) and locus coeruleus (El-Etri et al., 1999). Through verification of the stimulation positions, however, these structures were determined to be sufficiently distal to our stimulating electrode, and activation of them can be excluded as a likely confound (Figures 1B,C). On the other hand, one structure that may not be excluded is the magnocellular preoptic nucleus (MCPO), which is adjacent to the caudal HDB. For those cases, HDB/MCPO is treated as a unique structure, since MCPO is a major GABAergic projection to the MOB (Gracia-Llanes et al., 2010). By taking into account the small size of VDB and HDB in mice, we assume our stimulations were effective in both structures. Moreover, the stimulations were also effective for its subgroups, such as cholinergic (Macrides et al., 1981; Mesulam et al., 1983), GABAergic (Gracia-Llanes et al., 2010) and possibly glutamate neurons (Hur and Zaborszky, 2005) and local interneurons (Zaborszky and Duque, 2000). It is likely that a biased stimulation on the cholinergic/GABAergic neurons within VDB/HDB/MCPO might contribute to the heterogeneous responses in the MOB (Figures 2, 3, 4).

### NDB enhances M/T cell synchronization

Bursting is considered a more reliable mechanism for synaptic transmission than single spikes (Lisman, 1997). M/T cells all discharge spontaneously, exhibiting rhythmically bursting activity. For some units, the bursting is synchronized, and time locked with respiration (Figure 3A). It was also evident that rhythmic spiking is correlated with respiration-gated LFPs (Cenier et al., 2009). Interestingly, all units regardless of oscillation, demonstrated a shift of ISI to a spectrum of 10–25 ms, which falls into the gamma band of odor-induced oscillations (Cenier et al., 2009). The synchronization can be facilitated during odorant stimulation (Buonviso et al., 1992, 2006). With a more delicate experimental design, the spiking in an oscillatory circle was shown to precisely couple with odorant presentation (Cury and Uchida, 2010; Shusterman et al., 2011). The bursting frequencies coupled with respiration determine the temporal constraint for odor representation. Hence, an enhanced oscillation pattern (Figures 3, 4) might lead to a more precise odorant representation. More recent studies of M/T cell function for odor encoding have focused on the early events in a bout of odorants, since a short sampling is enough for odor identification (Schaefer and Margrie, 2007; Cury and Uchida, 2010; Carey and Wachowiak, 2011), albeit other mechanisms may be applicable (Gschwend et al., 2012). The duration of the NDB effect can suffice to accommodate this process (2–35 s vs. 50–150 ms). Conceivably, an increase of spiking frequency may cause an alteration of refractory period to delay M/T cell responses to odorants, and the latency *per se* is a way for M/T cells to encode odorants (Schaefer and Margrie, 2007). In contrast inhibition of M/T cell spikes can constrain the M/T cell response time window. Furthermore, synchronization implies an assembly of cooperating neurons (Engel et al., 1999), which suggest its role in higher brain function.

### How does NDB modulate MOB local circuitry?

Our work indicates that scopolamine blocked or attenuated the NDB potentiation in M/T cells, which reinforces the concept that mAChRs are required for NDB modulation of M/T cells (Inokuchi et al., 1987). *In vivo* work in animals with a comparable age also supports that muscarinic receptor activation facilitates M/T cell activity via reducing interneuronal activity (Elaagouby et al., 1991), even though it appears to contradict other previous findings. First, activation of cholinergic neurons using optogenetics inhibits all major types of neurons in the MOB (Ma and Luo, 2012). Given the non-specificity of electrical stimulation, the NDB potentiation might be caused by activation of GABAergic neurons, whereas the NDB depression might be caused by activation of cholinergic neurons. Since GABAergic neurons are more numerous in the basal forebrain than cholinergic neurons (Sarter and Bruno, 2002), GABAergic neurons might be more likely to be activated. It is understandable that, for NDB depression, activation of muscarinic receptors potentiates GABAergic interneuron activity which in turn increases GABA release and inhibits M/T cells (Castillo et al., 1999; Ghatpande et al., 2006; Pressler et al., 2007). Secondly, an *in vitro* study in younger animals showed that activation of nicotinic receptors but not muscarinic receptors induces potentiation of M/T cell activity (Castillo et al., 1999). This does not exclude activation of a subgroup of heterogeneously distributed cholinergic interneurons (Krosnowski et al., 2012). Additionally, short axon cells may be involved in NDB potentiation as they appear to have cholinergic endings (Kasa et al., 1995). Moreover, activation of GABAergic neurons by HDB stimulation might inhibit GABAergic granule cells through GC-M/T cell dendrodendritic disinhibition (Kunze et al., 1992; Gracia-Llanes et al., 2010), which leads to M/T cell excitation. In these cases, the NDB potentiation may not be sensitive to scopolamine (Figure 9).

On the other hand, the HDB might have an impact on M/T cell activity through PG cells located in the superficial glomerular layer (Castillo et al., 1999). These cells are GABAergic neurons, which form synapses with apical dendrites of M/T cells (Pirez and Wachowiak, 2008). Specifically, there is a unique subset of PG cells, or type I PGs, which are the only subclass of PGs innervated by olfactory nerves (Toida et al., 2000). They are GABAergic, and are likely targets of NDB GABAergic axonal terminals (Pignatelli and Belluzzi, 2008; Gracia-Llanes et al., 2010). It is also evident that muscarinic activation of dopaminergic PG cells can reduce their inhibition of M/T cells, which in turn drives excitation in M/T cells (Pignatelli and Belluzzi, 2008). Additionally, M/T neurons may also be activated directly via activation of nicotine (α 3β 4) receptors which results in a feedback GABAergic inhibition of M/T cells (D'Souza and Vijayaraghavan, 2012), but this might be the underlying mechanism for NDB-induced suppression (Figure 6).

mAChR-mediated long-lasting modulation of M/T cell spiking is consistent with *in vitro* recordings, where bath application of carbachol, a broad spectrum acetylcholine agonist, induces mitral cell discharge for several minutes (Castillo et al., 1999). This might work through GC-M/T cell interactions, as M/T cells form dendrodendritic synapses with GCs where excited M/T cells release glutamate to activate GCs, which results in GCs inhibiting M/T cells. NDB stimulation might suppress GC excitation, thereby disrupting the process of M/T cell-GC dendrodendritic inhibition, and generating long-lasting M/T cell excitation. Additionally, presynaptic cholinergic action facilitates the release of glutamate from M/T cells (Ghatpande and Gelperin, 2009), which auto-excites glutamate receptors on their dendrites, and further makes the excitation last for several seconds to several dozens of seconds.

We have used two complimentary drug delivery approaches since both of them have advantages and disadvantages (Figures 7–9). Presumably, dripping scopolamine was only effective locally in superficial MOB layers, blocking cholinergic synaptic transmission from local as well as from NDB cholinergic neurons, while systemic administration would provide a more global effect throughout the brain. Although the dripped drug could eventually travel beyond the MOB, it would diffuse further in the brain, and its impact would diminish significantly. Thus, scopolamine applied by dripping might not be able to attenuate cholinergic activity of the piriform cortex (pPC) driven by HDB (Zaborszky et al., 1986; Zimmer et al., 1999). In this situation, activation of the NDB-pPC-MOB circuitry might not be disrupted, and M/T cell spike rate potentiation shows not affected by scopolamine (Figure 9). Exactly how MOB circuitry is involved in NDB modulation requires further investigation.

### The impact of NDB modulation to MOB odor coding and beyond

The NDB is a major source of cholingergic/GABAergic projections to the neocortex, and plays a role in arousal and attention processing (Sarter and Bruno, 2000, 2002). It has been revealed that both nAChRs and mAChRs are involved in M/T cell responses to odorants (Chaudhury et al., 2009). NDB neurons also respond to odorants via the MOB (Linster and Hasselmo, 2000), with a slightly longer delay as compared with that of the MOB output neurons. The NDB-mediated topdown control of MOB principal cells may experientially modify responses in the MOB. Interestingly, the animal behavior state regulates GC-M/T cell dendrodendritic activity (Tsuno et al., 2008). The detection of a novel target with odor identity can be considered “attention.” Therefore, we propose that attention relevant clues might trigger NDB potentiation or suppression, thereby priming the principal neurons in anticipation of odorants. This effect might impact vision or audition as the rhythm in the MOB has been suggested to play a role in cross modal amplification and attentional selection (Schroeder et al., 2010). The proposed function of the HDB is thus considered as an “alert” center, keeping the animal's nose in a vigilant state. In this sense, the NDB potentiation/suppression can be considered a cellular substrate underlying olfactory “attention,” which is achieved by adjusting the M/T cell signal-to-noise ratio and the response time window for precise signal processing. Presumably, such a hypothesis of NDB modulation suggests its role in odor discrimination (Chaudhury et al., 2009), short-term memory (Ravel et al., 1994), and further perceptual learning (Fletcher and Chen, 2010). Furthermore, the NDB enhanced synchronization might have a global impact in the cortex that could be responsible for the involvement of the basal forebrain in Alzheimer's disease (Grothe et al., 2010).

## Author contributions

All experiments were performed at UMB. Xiping Zhan designed the experiments, Xiping Zhan and Pingbo Yin analyzed the data, Xiping Zhan and Thomas Heinbockel wrote the manuscript. All authors approved the final version.

### Conflict of interest statement

The authors declare that the research was conducted in the absence of any commercial or financial relationships that could be construed as a potential conflict of interest.

## Abbreviations

AC: anterior commissural connection
GC: granule cell
HDB: horizontal diagonal band
IP: intraperitoneally
ISI: interspike interval
LFP: local field potential
LV: lateral ventricle
mAChR: muscarinic acetylcholine receptor
MOB: main olfactory bulb
MCPO: magnocellular preoptic nucleus
M/T: mitral/tufted cell
nAChR: nicotinic acetylcholine receptor
NDB: nucleus of the diagonal band
ORN: olfactory receptor neurons
PG: periglomerular cell
pPC: piriform cortex
VDB: vertical diagonal band.
